# Health professionals’ attitudes toward religiosity and spirituality: a NERSH Data Pool based on 23 surveys from six continents

**DOI:** 10.12688/f1000research.52512.2

**Published:** 2021-09-21

**Authors:** Alex Kørup, Jens Søndergaard, Nada A Alyousefi, Giancarlo Lucchetti, Klaus Baumann, Eunmi Lee, Azimatul Karimah, Parameshwaran Ramakrishnan, Eckhard Frick, Arndt Büssing, Esther Schouten, Wyatt Butcher, René Hefti, Inga Wermuth, Rocio de Diego-Cordero, Maria Cecilia Menegatti-Chequini, Niels Christian Hvidt

**Affiliations:** 1 Research Unit of General Practice, University of Southern Denmark, Odense, 5000, Denmark; 2 Department of Mental Health Kolding-Vejle, University of Southern Denmark, Vejle, Region of Southern Denmark, 7000, Denmark; 3 College of Medicine, King Saud University, Riyadh, 11461, Saudi Arabia; 4 Department of Medicine, Federal University of Juiz de Fora, Juiz de Fora, Brazil; 5 Faculty of Theology, Albert-Ludwig-University, Freiburg, D-79085, Germany; 6 Center for Social Cohesion, Catholic University of Daegu, Daegu, South Korea; 7 Department of Psychiatry, Airlangga University, Surabaya, Indonesia; 8 Graduate Theological Union, University of California, Berkeley, Berkeley, USA; 9 Department of Psychosomatic Medicine and Psychotherapy, Technical University of Munich, Munich, 81675, Germany; 10 Munich School of Philosophy, Munich, 80539, Germany; 11 Institute of Integrative Medicine, University Witten/Herdecke, Herdecke, 58313, Germany; 12 Department of Neonatology, University Hospital Munich, Munich, 80366, Germany; 13 School of Divinity, King’s College, University of Aberdeen, Aberdeen, UK; 14 Research Institute for Spirituality and Health, University of Bern, Bern, Switzerland; 15 Department of Child and Adolescent Psychiatry, Psychosomatics and Psychotherapy, University Hospital Munich, Munich, 80336, Germany; 16 Research Group CTS 969 Innovation in Health Care and Social Determinants of Health, University of Seville, Seville, 41009, Spain; 17 Department and Institute of Psychiatry, University of São Paulo, São Paulo, 05403-010, Brazil; 18 Academy of Geriatric Cancer Research (AgeCare), Odense University Hospital, Odense, 5000, Denmark

**Keywords:** Health professionals, Religion, Spirituality, Data pool, International collaboration

## Abstract

Background

In order to facilitate better international and cross-cultural comparisons of health professionals (HPs) attitudes towards Religiosity and/or Spirituality (R/S) using individual participant data meta-analysis we updated the NERSH Data Pool.

Methods

We performed both a network search, a citation search and systematic literature searches to find new surveys.

Results

We found six new surveys (N=1,068), and the complete data pool ended up comprising 7,323 observations, including 4,070 females and 3,253 males. Most physicians (83%, N=3,700) believed that R/S had “some” influence on their patients’ health (CI95%) (81.8%–84.2%). Similarly, nurses (94%, N=1,020) shared such a belief (92.5%–95.5%). Across all samples 649 (16%; 14.9%–17.1%) physicians reported to have undergone formal R/S-training, compared with nurses where this was 264 (23%; 20.6%–25.4%).

Conclusions

Preliminary analysis indicates that HPs believe R/S to be important for patient health but lack formal R/S-training. Findings are discussed. We find the data pool suitable as a base for future cross-cultural comparisons using individual participant data meta-analysis.

## Introduction

Within the research field “R/S and health” several traditional meta-analyses have been published based on both cross-sectional, mixed or longitudinal studies showing varying results although mainly showing a positive correlation. ^1^ A recent meta-analysis by Garssen *et al*. based on longitudinal studies ^2^ also reported a marginal positive association between distinct aspects of religiosity (i.e., participation in public religious activities and importance of religion) and mental health, albeit religiosity was only able to explain 0.6% of the variation in mental health. The limited effect found, and the study conclusion, were later questioned, in part, due to the chosen definition of mental health. ^3^ A further problem was the heterogeneity of indicators of ‘religiosity’. A meta-analysis by Hackney *et al*. based on 34 cross-sectional studies on the relationship between different indicators of religiosity and mental health found religiosity to account for only 1% of the variation in mental health ( *r* = 0.1). ^4^ The latter meta-analysis also demonstrated that simply by adjusting the definitions of religiosity and psychological adjustment they were able to get a result that either supported a positive, negative or no relationship at all. ^4^ Both studies were thoroughly executed and are simply brought to attention here in order to exemplify two important challenges our research field must overcome. The first is the need to continually strive for the improvement of our scientific methods and tools. Below we will argue for a wider usage of meta-analyses based on individual participant data (IPDMA) as one way of mitigating some of the limitations of traditional meta-analysis. Second, we fear that continually separate efforts will be fruitless if researchers fail to develop common conceptual definitions of key concepts within R/S research, and instead keep proposing own definitions and instruments whose face validity are often limited to a local population. We cannot claim to offer a solution to this latter problem, but we argue below for the advantages we ourselves experience through our international and cross-cultural collaboration network.

The aim of this article is to 1) present how international data pooling can enable gold standard research methods like IPDMAs, 2) report the results of a newly updated network, citation and literature search leading to the 3 ^rd^ version of the NERSH Data Pool, 3) describe characteristics of the data pool, and descriptive statistics of observations’ demographics, 4) report selected details about the physicians and nurses in the data pool regarding their attitudes towards the influence of R/S on patient health, and their degree of training in handling R/S in medicine, and finally 5) to share ideas for future cross-cultural projects.

## Background

One important part of R/S in healthcare concerns the R/S of the health professionals delivering care, and their attitudes toward R/S in clinical practice. Quantitative measurement and cross-cultural comparisons of human values and attitudes are notoriously cumbersome and debated, ^5^ and probably especially so when the topic of interest lies at the nexus of religiosity, spirituality and health. ^1, 6^

Migration and population growth continually change the landscapes of cultures and faiths of the world’s countries ^7^ creating new demands of healthcare systems that historically were developed to function within a single belief system ( *i.e.* Christian Samaritanism *etc.*). This creates a need for a cross-cultural understanding and adds another challenging factor to this research field, requiring the highest levels of data quality and integrity to limit information bias and optimize statistical measurements.

Comparing research findings have hitherto been difficult because of limited comparability of study designs and study outcome, as described by Garssen *et al.* in their meta-analysis of R/S and mental health where they had to exclude 100 out of 181 eligible studies based on either methodological issues or incompatible study design or outcome measures. These differences are known throughout this research field and have made data pooling less feasible. Opportunities are missed and statistical analyses of greater precision are left unexplored.

Current differences build upon a history where only two decades ago, research within R/S in healthcare was almost solely based on populations of developed countries, and predominantly adhering to Christian worldviews. Although still dominated by North American and European research, ^1^ today this research field has roots in countries from all parts of the globe spanning six continents with all major faiths and spiritual orientations. The expansion of this field into other cultures and worldviews has created a more mosaic and complex picture of R/S in medicine world-wide.

Despite national and cultural differences, we believe a common denominator of human existence exists through which we experience and use R/S or secularism, and we find it important to take steps toward distilling those common characteristics within medical care that relate to HPs’ R/S. We believe a small step in that direction is to openly and respectfully share data and exchange ideas, as is the purpose of the NERSH Data Pool.

It is our experience that research communities in general have become better at exchanging experiences and sharing research data. The increased interest has been seen in the amount of publications mentioning ‘data sharing’ in their abstract over the last four decades from 46 in 1980 to 5,960 in 2019; ^8^ and this is with good reason, as sharing and pooling research data have been linked to higher research quality, ^9^ and have been recognized to forge fruitful research collaborations. ^9, 10^

Underlining the arguments for sharing and pooling research data, the current Covid-19 pandemic has shown us, on a historical scale, just how interconnected countries, and cultures, have become, bringing forth the imperative of healthcare systems to support not only cross-cultural care and understanding, but also prioritize international cooperation including sharing and pooling of data.

## Methods

In 2015, our international research collaborative Network of Research in Spirituality and Health ( NERSH.org) decided to build a large global data pool of health professionals’ (HPs) religiosity and/or spirituality (R/S) based on two pre-selected questionnaires. ^1^ The establishment and evolvement of the NERSH collaboration including the NERSH questionnaire have been described elsewhere, ^11^ as are two prior releases of the data pool. ^12, 13^ The following describes the method of updating this data pool with datasets made available after the release of the 2nd version of the data pool.

### Systematic searches

Eligible surveys were found using a combination of a network search (the NERSH collaboration), and both a citation search and a systematic literature review.

The network search utilized the global collaboration of researchers in NERSH. Past, on-going or planned surveys using either the Religion and Spirituality in Medicine, Perspectives of Physicians (RSMPP) questionnaire ^14^ or the NERSH Questionnaire are the topic of frequent correspondence between collaborators. The NERSH Questionnaire being basically RSMPP with several additions including support for the DUREL index. Also, several research groups have joined the collaboration with a priority to share their survey data once collected and published locally. Local restrictions may apply so that survey data are not released to be included into the data pool until certain criteria are met, why included data are not necessarily added in chronological order.

Citation and literature searches were performed by the first author in the period of January to February 2020. For the citation search we looked up citations in Web of Science referencing eight articles on the original RSMPP-survey published by Curlin in the period 2005 to 2008. ^14- 21^ The eight articles by Curlin used in the citation search were selected by Hvidt and Kørup in 2016 for the first systematic search. It was important for us to use the exact same approach in this study. Articles were chosen because they all rest on the RSMPP and were the first articles published by Curlin in the years after the RSMPP was published. Looking for publications using the RSMPP we judged it unlikely that at least one of these articles would not be referenced. All citing articles were reviewed on abstract level, and if the data source was the RSMPP, or the data source was unclear based on the title or abstract, the entire article was screened. To ensure we found all surveys based on the RSMPP, and to also find potential surveys based on the more recent NERSH Questionnaire, our search strategy also included a literature search in Google Scholar, Web of Science, Embase, Medline and PsychInfo using the search strings in Table 1. Survey data already included in the data pool were ignored. All searches were limited to English productions, and as the previous version of the data pool was based on the same search strategy in 2016, we only assessed publications from the year 2016 and forth.

**Table 1.** Citation search and literature searches performed January-February 2020. Results limited to publication year 2016 and later.

Citation search in Web of Science	Found articles
Curlin, F.A.; Lantos, J.D.; Roach, C.J.; Sellergren, S.A.; Chin, M.H. Religious characteris-tics of U.S. physicians: a national survey. J. Gen. Intern. Med. 2005, 20, 629-634.	129
Curlin, F.A.; Chin, M.H.; Sellergren, S.A.; Roach, C.J.; Lantos, J.D. The Association of Physicians' Religious Characteristics with their Attitudes and Self-reported Behaviors re-garding Religion and Spirituality in the Clinical Encounter. Med. Care 2006, 44, 446-453.	107
Curlin, F.A.; Dugdale, L.S.; Lantos, J.D.; Chin, M.H. Do religious physicians dispropor-tionately care for the underserved? Ann. Fam. Med. 2007, 5, 353-360.	34
Curlin, F.A.; Lawrence, R.E.; Chin, M.H.; Lantos, J.D. Religion, Conscience, and Contro-versial Clinical Practices. The New England Journal of Medicine 2007, 356, 593-600.	200
Curlin, F.A.; Lawrence, R.E.; Odell, S.; Chin, M.H.; Lantos, J.D.; Koenig, H.G.; Meador, K.G. Religion, spirituality, and medicine: psychiatrists' and other physicians' differing ob-servations, interpretations, and clinical approaches. Am. J. Psychiatry 2007, 164, 1825-1831.	94
Curlin, F.A.; Odell, S.V.; Lawrence, R.E.; Chin, M.H.; Lantos, J.D.; Meador, K.G.; Koenig, H.G. The relationship between psychiatry and religion among U.S. physicians. Psychiatr. Serv. 2007, 58, 1193-1198.	66
Curlin, F.A.; Sellergren, S.A.; Lantos, J.D.; Chin, M.H. Physicians' Observations and Inter-pretations of the Influence of Religion and Spirituality on Health. Arch. Intern. Med. 2007, 167, 649-654.	72
Curlin, F.A.; Nwodim, C.; Vance, J.L.; Chin, M.H.; Lantos, J.D. To die, to sleep: US physicians' religious and other objections to physician-assisted suicide, terminal sedation, and withdrawal of life support. Am. J. Hosp. Palliat. Care 2008, 25, 112-120.	61
**Literature search**	**Found articles**
Google Scholar *"Religion and Spirituality in Medicine: Physicians’ Perspectives"*	33
Web of Science “TOPIC:(((questionn* OR survey* OR cross-section* OR national sample*) AND (religious OR religio* OR spiritual* OR religiosity) near/3 (professional* OR physician* OR psychiatris* OR doctor* OR staff* OR ((nurs* or medic*) near/3 (professor*)))))”	421
Embase + Embase Classic (Ovid®) “(((questionn* or survey* or cross-section* or national sample*) and (religious or religio* or spiritual* or re-ligiosity)) adj3 (professional* or physician* or psychi-atris* or doctor* or staff* or ((nurs* or medic*) adj3 professor*))).mp. [mp=title, abstract, heading word, drug trade name, original title, device manufacturer, drug manufacturer, device trade name, keyword]”	2,036
Medline (Ovid®) “(((questionn* or survey* or cross-section* or national sample*) and (religious or religio* or spiritual* or re-ligiosity)) adj3 (professional* or physician* or psychi-atris* or doctor* or staff* or ((nurs* or medic*) adj3 professor*))).mp. [mp=title, abstract, original title, name of substance word, subject heading word, key-word heading word, protocol supplementary concept word, rare disease supplementary concept word, unique identifier]”	1,348
PsychInfo (Ovid®) “(((questionn* or survey* or cross-section* or national sample*) and (religious or religio* or spiritual* or re-ligiosity)) adj3 (professional* or physician* or psychi-atris* or doctor* or staff* or ((nurs* or medic*) adj3 professor*))).mp. [mp=title, abstract, heading word, table of contents, key concepts, original title, tests & measures]”	1,091

### Inclusion and exclusion criteria

We only included data on health professionals based on either of the two questionnaires: Religion and Spirituality in Medicine, Perspectives of Physicians (RSMPP) ^14^ or NERSH Questionnaire, ^11^ the latter being a further development of the first. Customized versions were accepted if they were mainly the RSMPP or NERSH Questionnaire. Exclusion criteria were: Observations that were missing information on gender or only contained empty answers were excluded. Also, We also excluded observations who had entered we set a minimum an age of less than 18 years.  

### Building the NERSH Data Pool

The data pool versions were created consecutively upon earlier releases. Thus, only new datasets not already included in the 2nd version were added to the 3rd version. All importations were based on original raw datasets sent to us by the local researchers. Data were sent to us in various data formats (Stata, RRID:SCR_012763; IBM SPSS, RRID:SCR_002865; or Microsoft Excel, RRID:SCR_016137), and all were converted into Stata datasets (.dta) before import. For an open-access alternative to the statistical software the R Project for Statistical Computing (RRID:SCR_001905) can perform equivalent analyses. In case interpretation of the raw data was not straightforward, or vital information could not be extracted from published articles, the relevant researchers were contacted by e-mail or phone until the issue was resolved.

The data pool was created to comprise a total of 98 variables, of which 76 stem from the RSMPP, two are part of the DUREL-index not included (in complete form) in the RSMPP, and the remaining 20 variables are calculated variables aiding study categorization and usage of the included scales.

A codebook was created documenting available original and calculated variables and scales. ^22^

### Data security and ethical considerations

All observations in the data pool have been anonymized. The data pool is physically located in a secure server environment in Odense, Region of Southern Denmark, and containing only anonymized data upholds the latest security requirements of the General Data Protection Regulation (GDPR) of the European Union. The project was approved by the University of Southern Denmark Research & Innovation Organization (reg.nr: 10.312).

## Results

### Systematic searches

For the network search, knowledge about past or on-going research projects within the NERSH group were assessed. We knew from an earlier query that Baumann and Lee were in possession of survey data from 138 German chaplains using the NERSH Questionnaire from 2012 to 2014, and that their data had now been approved to be included into the NERSH Data Pool. ^23^ Also, a Swiss data sample of 104 general practitioners from a survey by Hefti *et al.* in 2015 was now available. ^24^ The Swiss survey was published in German language and would thus not have been found by the citation or literature searches.

The eight citation searches performed found a total of 763 hits including many duplicates. References were screened by the first author who identified three eligible surveys that were based on parts of the original RSMPP. A survey by Cordero *et al.* from Seville, Spain, examined 75 graduate students (nurses, podiatrists and physiotherapists) using the RSMPP and the DUREL index in 2017, ^25^ and the same research group also examined Portuguese nursing students in 2016 using the same questionnaires. ^26^ In addition, Menegatti-Chequini *et al.* in performed two surveys of psychiatrists in 2013–2014, a local facility sample in São Paulo (N = 84), and a nation-wide sample among members of the Brazilian Psychiatric Association (N=508) using a questionnaire based on the RSMPP. ^27, 28^ Details of the citation search hits are found in Table 1.

**Table 2.** Study name, country, age, gender and occupational characteristics of respondents.

			Age		Gender		Occupation							
Author/study*	Country	Sample year	Mean	SD	Female (%)	Male (%)	Physician**	Mid-wife	Nursing	Psychologist	Other therapist	Chaplain	Student	Other
Curlin, 2005	USA	2002	49.0	8.3	300 (26)	842 (74)	1,142	0	0	0	0	0	0	0
Schouten-Wermuth, 2016 ^†^	Germany	2014	38.9	10.3	1,398 (88)	195 (12)	515	286	636	18	1	0	0	46
Kuseyri, 2016 ^†^	Germany	2016	33.4	8.4	79 (66)	41 (34)	73	0	9	0	2	0	10	9
Hvidt-Frick, 2016	Germany	2014	34.8	11.4	132 (71)	53 (29)	48	0	125	0	0	5	0	6
Büssing, 2014 ^††^	Austria	2014	39.7	11.0	132 (71)	53 (29)	28	0	113	0	0	0	0	28
van Randwijk, 2018	Denmark	2012	48.9	12.5	387 (42)	524 (56)	911	0	0	0	0	0	0	0
Lee, 2013	Germany	2011	39.9	10.8	252 (63)	145 (37)	121	0	160	32	41	0	0	32
Al-Yousefi, 2012	Saudi Arabia	2010	36.6	9.2	97 (43)	128 (57)	225	0	0	0	0	0	0	0
Tomasso, 2011	Brazil	2010	31.5	8.7	132 (90)	14 (10)	0	0	146	0	0	0	0	0
Münger, 2017 ^†^	Switzerland	2016	54.4	9.7	25 (32)	54 (68)	79	0	0	0	0	0	0	0
Butcher, 2013 ^†^	New Zealand	2012	n/a***		39 (35)	73 (65)	112	0	0	0	0	0	0	0
Ramakrishnan, 2014	India	2012	32.5	10.8	161 (57)	121 (43)	282	0	0	0	0	0	0	0
Ramakrishnan, 2014	Indonesia	2010	29.2	3.8	65 (54)	55 (46)	120	0	0	0	0	0	0	0
Mukwayakala, 2018 ^†^	Congo	2012	35.2	7.9	28 (25)	84 (75)	112	0	0	0	0	0	0	0
Lucchetti, 2016	Brazil	2012	37.7	11.1	49 (25)	145 (75)	194	0	0	0	0	0	0	0
Lucchetti 2018 ^††^	Brazil	2018	28.5	3.4	102 (60)	69 (40)	171	0	0	0	0	0	0	0
Lee, 2019	South Korea	2015	34.0	9.4	194 (69)	87 (31)	42	0	130	28	1	0	0	0
Cordero, 2019	Spain	2018	28.0	6.9	48 (64)	27 (36)	0	0	0	0	0	0	75	0
Cordero, 2018	Portugal	2016	21.9	3.4	134 (85)	24 (15)	0	0	0	0	0	0	158	0
Lee, 2015	Germany	2014	54.2	7.5	48 (35)	90 (65)	0	0	0	0	0	138	0	0
Hefti, 2018	Switzerland	2014	53.8	9.7	31 (30)	74 (70)	105	0	0	0	0	0	0	0
Menegatti-Chequini, 2019	Brazil	2014	45.6	9.8	32 (38)	52 (62)	84	0	0	0	0	0	0	0
Menegatti-Chequini, 2016	Brazil	2014	48.4	11.9	205 (40)	303 (60)	508	0	0	0	0	0	0	0
Total			41.4	12.5	4,070 (56)	3,253 (44)	4,872 (68.6%)	286 (4.0%)	1,319 (18.6%)	78 (1.1%)	45 (0.6%)	143 (2.0%)	243 (3.4%)	121 (1.7%)

Note. * First publication of local sample if any. If not published the name of the head researcher and sample year is used. ** Broad definition of physician including MDs undergoing residency or specialty training. *** Age group of respondents available but not reported here.

† Thesis. †† Not published locally, sampling year used for identification.

The literature search resulted in a total of 4,929 hits. Restricting the search to articles published 2016 or later reduced the count to 1,133 hits. 20 articles were retrieved in full length but did not prove eligible. In summary, the literature search did not find any eligible surveys not already found by the network and citation searches.

In total, six new surveys were eligible to import into the NERSH Data Pool. The researchers were contacted and invited to submit their original data, and all agreed with written confirmation. The surveys varied from surveys sampled across an entire nation to surveys done at a single facility or hospital. The study with Spanish nursing students (Cordero, 2019) was sampled among graduate students from The Faculty of Nursing, Podiatry and Physiotherapy in Seville. ^25^ By the same research group, the Portuguese nursing students (Cordero, 2018) were 3 ^rd^- and 4 ^th^-year students from the School of Health of University of Algarve and School of Health of Polytechnic Institute of Santar. ^26^ The data on German hospital chaplains were collected in a nation-wide survey by Lee *et al.*, ^23^ while the Swiss sample of General practitioners was a random sample from the region of Bale and Aarau by Hefti *et al.* ^24^ From Menegatti-Chequini *et al.* we received two samples, the first based on a survey sent to members of the Brazilian Psychiatry Association (ABP) and an additional sample from a single psychiatric department Instituto de Psiquiatria, Hospital das Clínicas, Faculdade de Medicina, Universidade de São Paulo (IPqHC-FMUSP). Duplicates from the ABP and IPqHC-FMUSP samples were removed.

### Demographics

After applying exclusion criteria, a total of 1,068 unique observations were added to the NERSH Data Pool. The entire 3 ^rd^ version of the data pool ended up comprising 7,323 observations, including 4,070 females and 3,253 males. Mean age (SD) was 41.4 (12.5). Looking at the entire data pool response rates ranged from 18% (116 responses out of 642 questionnaires sent in New Zealand with no possibility for follow-up on non-responders) to 95% (Brazil) and 99% (Indonesia), the latter two secured due to tight follow-up including personal meetings and encouragements to complete the forms. Crude response rate was 54.7% for all currently included studies.

After inclusion of the found datasets the complete data pool comprised 4,872 physicians, 1,319 nurses, 286 midwives and other HP occupations in smaller numbers (see Table 2 for details). Medical specialties were categorized by major specialty groups ( Table 3). The largest group of specialties were gynecology/obstetrics with 1,788 participants mainly from the German sample from perinatal care professionals, followed by 1,591 working in psychiatry, 953 from Internal Medicine, 842 from general practice, 447 from surgical specialties, 236 within pediatric medicine, and 143 from paraclinical specialties ( *i.e.* laboratory sciences and supportive branches of medicine not directly involved patient care).

**Table 3.** Distribution of grouped medical specialties in the studies*. Students not included.

Study/Medical specialty	Medical	GP	Obst/ gyn	Surgical	Para-clinical	Pediatric	Psychiatry	Other	Total
Curlin, 2005	314	304	80	118	45	147	100	34	1,142
Schouten-Wermuth, 2016	0	0	1,593	0	0	0	0	0	1,593
Kuseyri, 2016	29	0	5	21	0	9	9	28	101
Hvidt-Frick, 2014	116	0	0	38	0	0	0	28	182
Büssing, 2014	66	0	0	37	0	0	0	60	163
van Randwijk, 2018	145	209	31	132	34	17	43	12	623
Lee, 2013	0	0	0	0	0	0	397	0	397
Al-Yousefi, 2012	70	73	31	30	0	21	0	0	225
Münger, 2017	0	79	0	0	0	0	0	0	79
Butcher, 2013	0	0	0	0	0	0	112	0	112
Ramakrishnan (India), 2014	17	49	11	9	50	11	45	33	225
Ramakrishnan (Indoensia), 2014	8	23	7	25	14	2	1	17	97
Lucchetti, 2016	146	0	10	26	0	12	0	0	194
Lucchetti, 2018	42	0	20	11	0	17	11	70	171
Lee, 2019	0	0	0	0	0	0	281	0	281
Hefti, 2018	0	105	0	0	0	0	0	0	105
Menegatti-Chequini, 2019	0	0	0	0	0	0	84	0	84
Menegatti-Chequini, 2016	0	0	0	0	0	0	508	0	508
Total	953 (15.2%)	842 (13.4%)	1,788 (28.5%)	447 (7.1%)	143 (2.3%)	236 (3.8%)	1,591 (25.3%)	282 (4.5%)	6,282 (100%)

*Note.* * The samples of Brazilian nurses (Tomasso, 2010) and physicians from Congo (Mukwayakala, 2018) contained no information about medical specialty. Also, medical specialty was not mandatory in all surveys, thus totals may differ from actual sample sizes.

Almost all health professionals supplied information about their religious affiliation (N = 7,158; 97.7%). If the responder did not want to supply this information the answer was treated as missing. All questionnaires had a “No affiliation” option, and some also included the options “None”, “Atheism” and/or “Agnosticism”. These options were all grouped together based on their common denominator of not being affiliated with a religion. Answers of religious affiliation were categorized in groups of the major faiths: Buddhism, Hinduism, Judaism, Mormonism, Islam and Christianity. Christian denomination ( *i.e.* Orthodoxy, Catholicism or Protestantism) was registered for samples providing this information. Answers that did not fit any of these groups were placed in the “Other” group.

Religious affiliations from predominantly Islamic cultures were almost entirely Muslim (Saudi Arabia 100% and Indonesia 86%), whereas Hinduism was predominant in the Indian sample, N = 195; 71%.

Looking at the entire data pool the largest group was Christian denominations (N = 4,189; 59%), represented in all samples apart from the sample from Saudi Arabia. The second largest was the group of responders that declared themselves not affiliated with a religion, atheist or agnostic (N = 1,529; 21%). Muslims comprised the third largest group in the data pool (N = 504; 7%) ( Table 4).

**Table 4.** Distribution of religious affiliations in the studies.

Study/Religious affiliation	No affiliation*	Buddhist	Hindu	Jewish	Mormon	Muslim	Christian	Other	Total
Curlin, 2005	114	12	53	181	17	33	636	79	1,125
Schouten-Wermuth, 2016	409	6	0	1	0	13	1,077	1	1,507
Kuseyri, 2016	22	0	0	0	0	78	1	1	102
Hvidt-Frick, 2014	52	0	0	0	0	0	123	8	183
Büssing, 2014	29	0	0	0	0	0	148	6	183
van Randwijk, 2018	185	2	1	0	0	6	688	17	903
Lee, 2013	122	7	0	0	0	5	244	19	397
Al-Yousefi, 2012	0	0	0	0	0	225	0	0	225
Tomasso, 2010	17	2	0	0	0	0	98	29	146
Münger, 2017	8	1	1	2	0	1	66	0	79
Butcher, 2013	53	3	4	0	0	0	40	4	106
Ramakrishnan (India), 2014	8	1	195	0	0	37	35	0	276
Ramakrishnan (Indonesia), 2014	0	0	2	0	0	103	15	0	120
Mukwayakala, 2018	0	0	0	0	0	0	100	0	100
Lucchetti, 2016	9	0	0	0	0	0	163	22	194
Lucchetti, 2018	25	0	0	0	0	0	117	27	169
Lee, 2019	151	36	0	0	0	0	93	1	281
Cordero, 2019	37	0	0	0	0	1	33	4	75
Cordero, 2018	71	2	0	0	0	0	68	14	155
Lee, 2015	0	0	0	0	0	0	138	0	138
Hefti, 2018	19	0	0	2	0	2	80	0	103
Menegatti-Chequini, 2019	35	0	0	0	0	0	33	15	83
Menegatti-Chequini, 2016	163	0	0	0	0	0	193	152	508
Total	1,529 (21.4%)	72 (1.0%)	256 (3.6%)	186 (2.6%)	17 (0.2%)	504 (7.0%)	4,189 (58.5%)	399 (5.6%)	7,158 (100%)

*Note.* * Including atheists and agnostics.

### Influence of R/S on patient health

**Figure 1.**
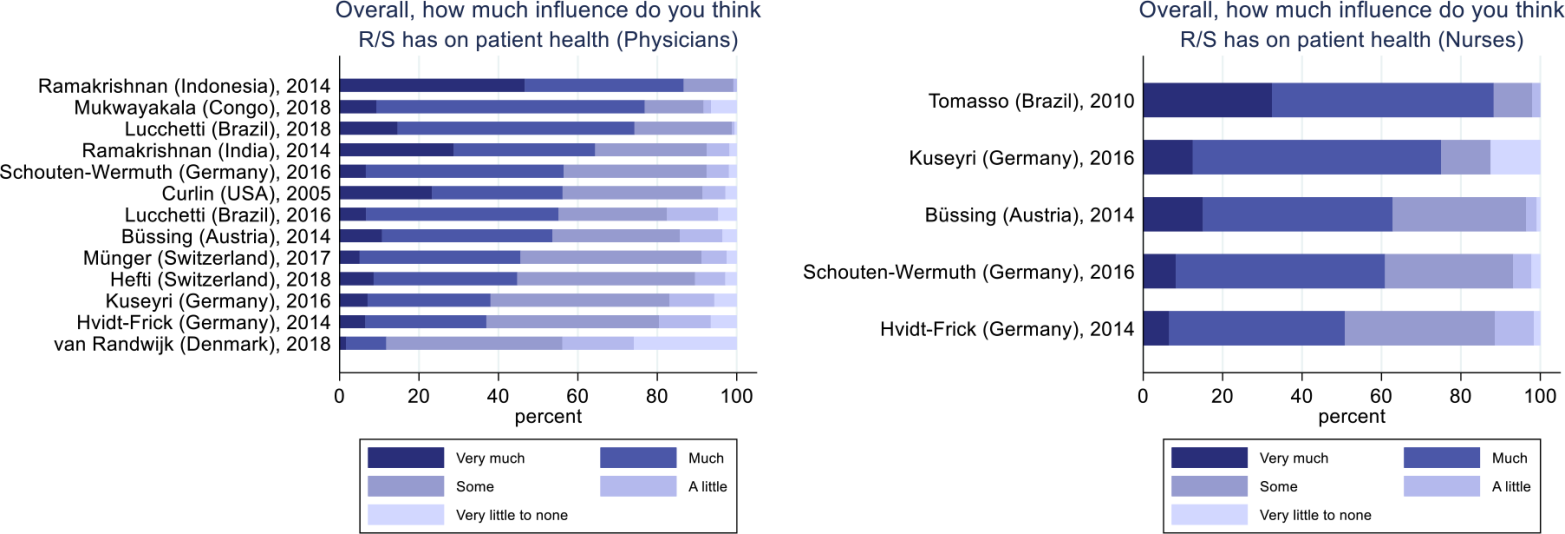
Physicians' and nurses' opinion about the influence of R/S on patient health. Physicians, N = 3,700. Nurses, N = 1,020.

Responders were asked about the potential influence of R/S on patient health in general. Across all samples 3,700 physicians answered this question, of which 1,767 (48%, CI95% 46.4%–49.6%) replied that R/S has “Much” or “Very much” influence on patient health ( Figure 1). The number of physicians believing that R/S has at least “Some” influence on patient health was 3,078 (83%, 81.8%–84.2%). In comparison, for 1,020 nurses these proportions were 658 (65%, 62.1%–67.9%) and 955 (94%, 92.5%–95.5%) respectively. Due to the large between-group heterogeneity, statistical significance test of differences was not feasible.

### R/S Training of HPs

**Figure 2.**
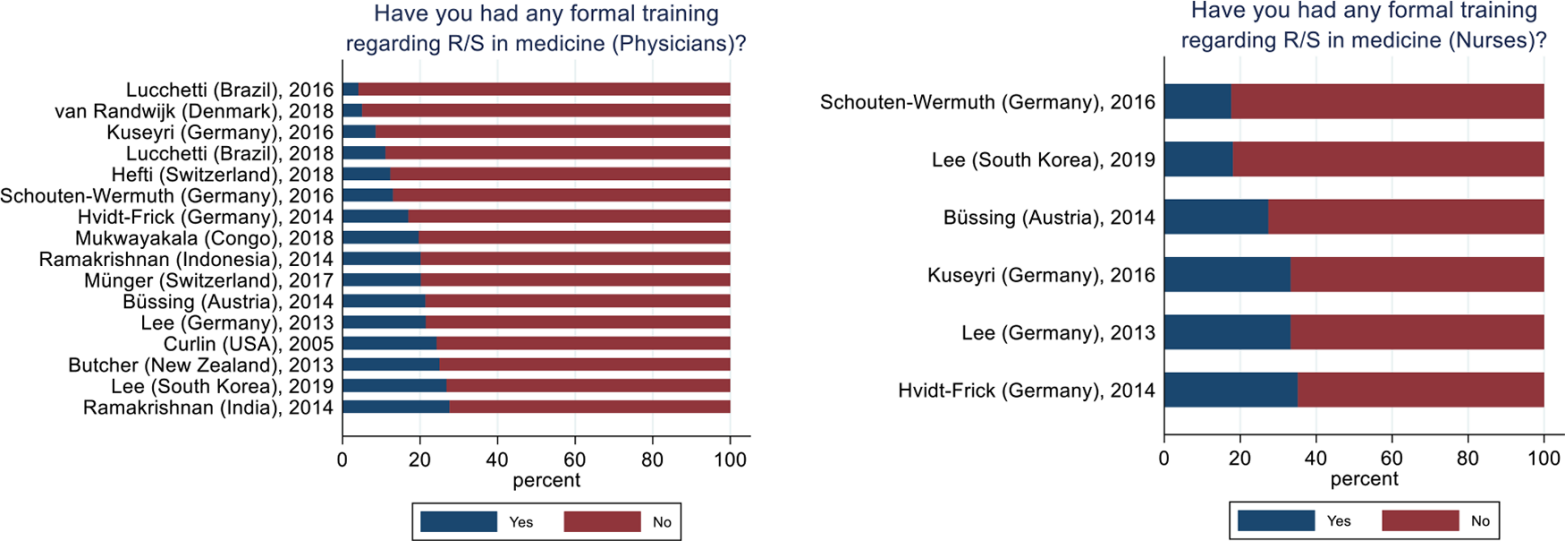
Physicians' and nurses' formal training in R/S. Physicians, N = 3,987. Nurses, N = 1,158.

Across all samples 649 (%, CI95%) (16%, 14.9%–17.1%) physicians reported to have undergone any formal training regarding R/S in medicine with the lowest proportion was 4% (Lucchetti, 2016) and the highest 28% (Ramakrishnan, India, 2014). For nurses this amounted to 264 (23%, 20.6%–25.4%) ranging from 18% (Schouten-Wermuth, 2016) to 35% (Hvidt-Frick, 2014) ( Figure 2).

## Discussion

Our discussion here will focus first on the reported statistics and general composition of the data pool, and second, we will discuss the objectives behind building the NERSH Data Pool.

### Data pool characteristics

Using both network, citation and systematic literature searches we found six new survey samples based on the RSMPP or NERSH Questionnaire, which were added to the preexisting second version of the data pool, now released as the NERSH Data Pool 3.0. Here we have presented the demographics of the complete data pool and select variables regarding attitudes toward influence of R/S on patient health, and whether responders had any formal training in R/S in medicine.

The included samples were collected in very distinct locations, some were collected from national organizations, and others were collected at single facilities. The cultures and religious landscapes of the represented countries differ, and participants' attitudes are likely affected thereof. Some samples include only HPs of a specific profession ( *i.e.* only physicians, nurses or chaplains), where others included a variety of health professions.

The heterogeneity between studies was expected as part of the design and is the reason why it did not make sense to test between-sample differences of descriptive variables. As expected, when looking at attitudes toward potential influence of R/S on patient health, we see large variations between studies, and based on the 95% confidence intervals nurses more often reported this influence on patient health than the physicians did. This is in line with earlier research on differences in nurses' and physicians' attitudes toward R/S, ^29, 30^ although we must underline that the crude descriptive findings reported here are at study-level and not controlled for within-study confounders. This was beyond the scope of this descriptive analysis of the data pool.

Also, the reported R/S training of the physicians and nurses in the data pool varied largely. Again, we read two important points out of this. 1) Physician and nurses are largely under-educated when it comes to handling R/S in the clinical setting ( *i.e.* 16% and 23% respectively had some education, leaving approximately four out of five physicians and nurses as having no formal education in this matter). We have no information about the degree or length of educations that were reported. 2) Large differences between sample may signify how the focus on R/S education local is influenced by local culture.

***MAs and IPDMAs***

Traditional meta-analyses (MAs) are the recommended approach when comparing results of several studies. MAs examining HPs’ attitudes in clinical practice are still scarce, and IPDMAs even more so.

In this study we have reported how we have built a pool of individual HP survey responses that enables us to perform IPDMA. Already a single recent IPDMA study compared the religiosity of physicians from seven countries, and their self-reported influence of their religiosity on their clinical practice. Religiosity and influence of religious beliefs were most pronounced in India, Indonesia, and a European faith-based hospital. Half (50%) of the physicians examined reported being influenced that their work as a physician was influenced by their own religiosity. ^31^ Using individual participant data, the authors were able to conduct a sensitivity analysis of potential confounders at sample level, and thus demonstrated the potential of this data pool.

Koenig *et al*. recently expressed detailed concerns about the biases introduced when using meta-analyses within R/S research. ^3^ They describe R/S and mental health research as a social science where meta-analyses should only be used to describe heterogeneity and not as much searching for consistency and generalizability of study findings across populations. Koenig *et al.* highlights several common critique points of MAs including when studies measure different variables, incomplete or unstandardized results, inability to account for inter-study variation, heterogeneity due to broad inclusion criteria without the possibility to limit the analysis to sub-groups within the samples, and susceptibility to the ecological fallacy. All these critique points are mitigated by the improved IPDMA-design, which we enable when collecting individual participant data. ^32^

Meta-analyses are considered one of the gold standards behind evidence-based health care, and the number of published meta-analyses have increased markedly over the last decades although average study quality has been questioned. ^33^ One of the caveats is that when collections of samples are very heterogeneous, comparisons of variables using a traditional meta-analysis design are likely to lead to biased results if within-sample confounders are not controlled for at the individual-level (i.e. ecological fallacy). ^34^ This fallacy is suspected to bias interpretations of meta-analyses where individual participant data were not available to the researchers, who thus had to rely on simple aggregated effect measures of the included studies ( *i.e.* at study-level). Sometimes effect measures even differ between the included studies, hence they must be converted to a common effect measure by meta-analysis researchers. This introduces yet another step in the meta-analysis and thus a risk for bias/error.

Koenig *et al.* also problematized the risks introduced when study results are reduced to a single value. ^3^ For the social and health sciences this is however a key concept that has driven scientific research to where it is today. This is not saying that this reductionism does not include biases and caveats that researchers must understand and respect in their interpretation of their results. Especially within psychometrics ( *i.e.* regarding R/S and mental health) measurements may lack the validity and reliability compared to measurements from more objective sciences. Still limited by these known biases, psychometrics have not only made quantifiable scientific comparisons possible within mental health research, but have also generated a wealth of crucial clinical instruments positively affecting lives and disease courses of patients, some examples are scales for measuring severity of mental illnesses during treatment like Hamilton Depression Rating Scale, ^35^ Global Assessment of Functioning, ^36^ IQ tests like WAIS-VI ^37^ and more. Measuring aspects of R/S is notoriously difficult, mostly due to the personal and subjective dimensions that are natural parts of R/S experiences. Challenges caused by imprecise measurements, and/or attempts to compare results from studies using different or custom instruments ( *i.e.* comparing apples and oranges) does not implicitly negate those instruments, but rather demonstrate a lack of collaboration amongst researchers of this field.

We thus believe, that the use of an instrument, or scientific method like meta-analyses, that over time has demonstrated its ability to advance health care and/or enrich the research thereof, should not be discontinued because of its imprecisions before another instrument or scientific method with improved characteristics are suggested to take its place.

***Assimilating research designs and measurements***

It has been argued that MAs are so complex that mistakes are inevitable. ^33^ We truly acknowledge the difficulty in conducting MAs, and most likely not a single MA is 100% perfect. Still, this is not an argument against using this method, because the argument of embedded error is applicable to practically most research methods including both quantitative and qualitative methods. Planning and deciding on research designs, deciding how to collect and filter the data, how strictly to enforce an interview guide, the handling of missing answers/observations, the weighing of pros and cons of different statistical strategies for analysis, choosing which statistics to report and how to interpret them. These are just some examples where research projects are susceptible to subjective decisions by human researchers that are prone to make mistakes and bad judgements. Rather than giving up on these methods we believe this calls for our continuing focus on our own human biases, and also for the need to systematize research methods using internationally recommended gold standards (like PRISMA) that precisely aims to document and limit these errors. ^38^ Also, instead of limiting research on R/S and health to theological and qualitative methods, we should welcome diverse and mixed research strategies, all adding valuable perspectives on a theoretical common consensus among researchers. This way we will continually equip ourselves with the latest and best instruments, with which we will attempt to prove or disprove the current hypotheses about how R/S is related to patient health and health care in general. Only this way can we make reason for decision makers to level the importance of R/S in health care with other central health topics crucial for patient health and well-being.

Like any other scientific research design, IPDMA has weaknesses, the largest being heterogeneity, which is largely caused by differences in sampling (location, culture, profession and sampling method), and differences in subjective judgements in the local samples.

Still, pooling survey data in the NERSH Data Pool will enable us to perform meta-analyses using the individual data of survey participants. In summary IPDMA carries both statistical and clinical advantages over regular traditional MA. ^32, 39^ Some advantages include: 1) the ability to utilize data from yet unpublished studies and or outcome measures thus reducing publication bias; 2) standardized statistical analysis across studies; 3) ability to perform analysis on sub-groups participants ( *i.e.* certain religious affiliations, occupations or medical specialties); 4) ensure consistent inclusion and exclusion criteria; 5) standardized handling of missing values across samples; and 6) overcoming the ecological fallacy of traditional meta-analysis by enabling analysis at the individual level. ^34^

## Conclusions

The above results and discussion highlight several important prerequisites for this research field, and we argue in favor of the following. 1) The psychometric constructs within R/S should be measured only with validated and broadly accessible instruments. 2) Ideally, researchers should stick to the same validated instruments. Even an average measure with known limitations, and used by everyone, is worth much more to this research field than a more precise measure used only by the few. 3) International and cross-cultural collaborations should be developed in order to bring researchers together. Our own experience from the NERSH-collaboration networking across national borders, and not least cultures, states and promotes a whole-hearted respect of each other’s worldviews, while cultivating an assimilated professional work ethic that demands the highest scientific standards. 4) We highly recommend sharing research data, in order to utilize statistical analysis of greater power at the individual level ( *i.e.* enabling IDPMA). We see the advantages of this within the niche of HPs R/S through the NERSH Data Pool, but data pooling like this could lift the entire research field of “R/S and health” and “Spiritual care” into a new era of scientific research.

The NERSH Data Pool of health professionals’ attitudes towards R/S in medicine is our attempt to help lift the quality of meta-analyses within this field. We have no knowledge about a similar data pool, and we look forward to test and retest hypotheses about R/S in medicine using its qualities.

## Limitations

Large between-samples heterogeneity was expected due to differences in sampling and culture of the background populations. Due to the reduced external validity, any attempt to compare local survey results must be done with caution and should control for within-sample confounders. Meta-analyses should use random-effects modeling.

We know from another project/article (currently undergoing peer-review elsewhere) that we can reduce the between-study heterogeneity by grouping the physicians by medical specialty. Further stratification of the health professionals in the data pool would likely further reduce the heterogeneity making comparisons more feasible. A disadvantage of this approach would be the risk of diluting a potential effect by creating small groups. However, the current article is not a meta-analysis, and we have chosen to report the complete content of the data pool here. Another and more long-term strategy to reduce heterogeneity would be to reduce methodological biases of sampling of future datasets. This could be done for instance by ensuring thorough cross-cultural validation of local questionnaires including qualitative feedback from health professionals who completed the questionnaire, ensuring that responders are presented with explaining definitions of central terms and concepts referenced in the questionnaire, and unifying the planning and execution of the local surveys internationally.

We have not been able to control for cohort effects because none of the samples have performed a follow-up survey.

The 2012 survey of Brazilian physicians ^40^ was based on interviews rather than self-administered questionnaires, which may have led respondents to give less extreme answers in fear of stigmatization. Contrary, it may be argued that face-to-face interviews limit acquiescence bias where responders tire out in written questionnaires and give the same answer to multiple subsequent questions.

## Perspectives

Researching and developing spiritual care at a national level is as important as ever, but it is not until we undertake the challenge of understanding international and cross-cultural differences that we can hope to truly develop our own culturally framed healthcare system. Also, we support that both researchers and health care stakeholders take candid and openhearted interest in healthcare systems from other cultures in order to allow a united and global growth in healthcare.

### Future work and clinical implications

In the near future, we will use the described data pool to test and retest hypotheses about R/S in medicine using IPDMA designs. Some planned analyses are: 1) the association of HPs’ attitudes and self-reported behavior regarding R/S in the clinical encounter; 2) R/S characteristics and attitudes of physicians from different medical specialties; and 3) Attitudes of HPs considering controversial ethical situations in healthcare.

We believe this work will better our understanding of how HPs values work in clinical practice, and aiding the development of R/S curricula that will help HPs learn how to incorporate spiritual care into their treatment of patients from any culture, and despite potentially differing world-views.

## Invitation to collaborate

Please contact last author (NCH) if you are interested in joining the NERSH collaboration.

## Data availability

### Extended data

Open Science Framework: Extended data for ‘Health professionals’ attitudes toward religiosity and spirituality: a NERSH Data Pool based on 23 surveys from six continents’. http://doi.org/10.17605/OSF.IO/J79PT. ^19^

This project contains the following extended data:

• NERSH Data Pool 3.0 Codebook. Excel file describing the structure, variables and scales included in the NERSH Data Pool 3.0. The file also includes an overview of the 23 samples included.

Data are available under the terms of the Creative Commons Zero “No rights reserved” data waiver (CC0 1.0 Public domain dedication).

## Footnotes

^1^
In this paper we will use R/S as a broad concept embracing both religiosity and spirituality. Religiosity being defined as the beliefs, practices and experiences pertaining to an organized religion or belief system; where spirituality is defined by a spectrum from evolved humanistic values through striving for an inner truth to contact with the transcendent. When we use them in unison here it is because this makes the most sensitive measure, and because we lack more specific definitions that are globally accepted. This is also the notation used in the original RSMPP questionnaire and the updated NERSH Questionnaire.
