# Supplementary material for: Health professionals’ attitudes toward religiosity and spirituality: a NERSH Data Pool based on 23 surveys from six continents
Source: F1000Res. 2021 Sep 21;10:446. Originally published 2021 Jun 4. [Version 2] doi: 10.12688/f1000research.52512.2 (PMC8607302; doi:10.12688/f1000research.52512.2)
Supplement: Peer review history

# Peer review: Reviewer response for version 2

**DOI:** 10.5256/f1000research.77416.r98197

**Published:** 2021-11-19

**Authors:** Fiona Gardner

**Affiliations:** 1 Social Work and Social Policy, Rural Health School, La Trobe University, Bendigo, VIC, Australia

This is an interesting article that follows up the NERSH advocacy for having a common data pool information to build comparisons and knowledge re: health professionals attitudes towards R/S. The network involved has expanded to include a wider diversity of religious backgrounds which increases its usefulness. From my perspective, the strength of the article is in its findings among health professionals of the relevance of R/S in treating people and the lack of training to help them do so. A limitation is the focus on medically trained professionals, not much nuance re: social workers, for example, compared to chaplains or occupational therapists.

 There are of course competing views about the value of this approach and these are considered about to some extent addressed - they do add they could welcome more mixed method strategies which would add nuance to the data. I think there remain issues about potentially missing information when you all use the same data set - especially when you try to move to a wider range of what R/S can mean. However, including some qualitative studies that build from the data set could balance that out. Increasing the number of people involved in non Christian and non Western parts of the world would also be helpful - which they acknowledge. It would be interesting for example to see where First Nations perspectives would fit. Overall, however, I do support indexing.

Is the work clearly and accurately presented and does it cite the current literature?

Yes

If applicable, is the statistical analysis and its interpretation appropriate?

I cannot comment. A qualified statistician is required.

Are all the source data underlying the results available to ensure full reproducibility?

Yes

Is the study design appropriate and is the work technically sound?

Yes

Are the conclusions drawn adequately supported by the results?

Yes

Are sufficient details of methods and analysis provided to allow replication by others?

Yes

Reviewer Expertise:

Religious/spirituality research in health care but primarily qualitative.

I confirm that I have read this submission and believe that I have an appropriate level of expertise to confirm that it is of an acceptable scientific standard.

# Peer review: Reviewer response for version 2

**DOI:** 10.5256/f1000research.77416.r94930

**Published:** 2021-11-01

**Authors:** Anja Visser

**Affiliations:** 1 Faculty of Theology and Religious Studies, Comparative Study of Religion, University of Groningen, Groningen, The Netherlands

I thank the authors for their careful consideration of my previous comments. Though I had envisioned a more thorough restructuring of the text with my comments, I think the purpose and procedures of the study have been sufficiently clarified and the discussion of the limitations sufficiently addresses my initial concerns.

Is the work clearly and accurately presented and does it cite the current literature?

Partly

If applicable, is the statistical analysis and its interpretation appropriate?

Yes

Are all the source data underlying the results available to ensure full reproducibility?

Yes

Is the study design appropriate and is the work technically sound?

Yes

Are the conclusions drawn adequately supported by the results?

Partly

Are sufficient details of methods and analysis provided to allow replication by others?

Yes

Reviewer Expertise:

Spiritual care, spirituality, coping, oncology

I confirm that I have read this submission and believe that I have an appropriate level of expertise to confirm that it is of an acceptable scientific standard.

# Peer review: Reviewer response for version 1

**DOI:** 10.5256/f1000research.55805.r89010

**Published:** 2021-08-02

**Authors:** Anja Visser

**Affiliations:** 1 Faculty of Theology and Religious Studies, Comparative Study of Religion, University of Groningen, Groningen, The Netherlands

Thank you for the invitation to review your paper, discussing the expansion of the NERSH database, its (potential) function and the attitudes toward and training in spiritual care of physicians and nurses in the database.

 To me, the most valuable point of this paper lies in the discussion of the potential of meta-analyses based in individual participant data (IPDMA). Discussion about the use of meta-analysis in psychology in general and in the psychology of religion in particular is becoming stronger and I think the authors offer an important avenue for mitigation of these issues with the suggestion to make more use of IPDMA and to build a database like the NERSH database to enable these types of studies. I would, therefore, recommend that the authors place more emphasis on this issue in their paper. For example, by moving this section of the discussion to the introduction section. This would also resolve the lack of clarity about the purpose of the paper that the introduction now suffers from. 

 At the same time, there is a weakness in the argumentation for the NERSH database as a 'good example' for IPDMA research, which is shown very clearly in the demonstration of the use of the database to examine the attitudes toward and training in spiritual care of physicians and nurses. As the authors correctly emphasize, IPDMA allows for a reduction of bias introduced in meta-analyses because of the heterogeneity between studies. However, even in the reasonably large dataset of NERSH, no statistical tests could be applied because of between-study heterogeneity (p. 8). A more thorough discussion of this point is needed in the paper, I think, if the authors really want to make a convincing argument for the use of databases such as NERSH: What is needed to reduce this heterogeneity, what might be disadvantages of a homogenizing approach and how much information is necessary to enable sufficient statistical power? The authors briefly discuss some of these issues on p13, but I recommend expanding this.

 Some more minor suggestions I have concerns of a lack of clarity about method.

- On p4, section 'building the NERSH Data Pool' it is stated that only survey samples that weren't already included were selected. However, I wondered if this meant that any repeated-measures samples would be excluded? Though I understood toward the end of the paper that such samples were non-existent, I suggest changing the word 'samples' to 'datasets', to avoid this misunderstanding.
- I also wasn't fully clear on the search strategy. Though I could follow the text, I became confused when looking at the upper half of Table 1; Have you looked for studies that cited those studies by Curlin et al.? If so, why did you choose these studies as for the citation search?  
- Do I understand it correctly that Table 2 contains all of the datasets currently in the NERSH database and that the ones listed on p7 are the new ones that were added to the third version? If so, please make this more clear in the text. 
- Why have you chosen to only examine the data of physicians and nurses?
- Have I understood correctly that the exclusion criteria mentioned on p8 are missing information on gender, only containing empty answers and not being 18years of age or older? Perhaps this could be made more clear in the methods section?

Finally, there are some misspellings and awkward sentences throughout the text that another careful reading should be able to get out. 

 Overall, I really appreciate the efforts of the authors to advance research methodology in the field of religion, spirituality, and health, and I think this paper helps the field make an important step in that direction..

Is the work clearly and accurately presented and does it cite the current literature?

Partly

If applicable, is the statistical analysis and its interpretation appropriate?

Yes

Are all the source data underlying the results available to ensure full reproducibility?

Yes

Is the study design appropriate and is the work technically sound?

Yes

Are the conclusions drawn adequately supported by the results?

Partly

Are sufficient details of methods and analysis provided to allow replication by others?

Yes

Reviewer Expertise:

spiritual care, spirituality, coping, oncology

I confirm that I have read this submission and believe that I have an appropriate level of expertise to confirm that it is of an acceptable scientific standard, however I have significant reservations, as outlined above.

# Response

**Published:** 2021-09-02

**Authors:** Alex Kørup

**Affiliations:** University of Southern Denmark, Denmark

Response to reviewer Anja Visser

 Thank you for reviewing our manuscript. Below I attend to all of your comments. The changes will be included in the 2nd version of the article uploaded along this response. This reply and the updated version of the article has been approved by all authors.

 Comment #1

 ”… Discussion about the use of meta-analysis in psychology in general and in the psychology of religion in particular is becoming stronger and I think the authors offer an important avenue for mitigation of these issues with the suggestion to make more use of IPDMA and to build a database like the NERSH database to enable these types of studies. I would, therefore, recommend that the authors place more emphasis on this issue in their paper. For example, by moving this section of the discussion to the introduction section. This would also resolve the lack of clarity about the purpose of the paper that the introduction now suffers from.”

 Response #1

 Thank you for pointing this out. We have moved the suggested section and strengthened the introduction section which are now more in line with the main purpose of the paper: To suggest, and present an example of, how data pooling can enable future IPDMA studies.

 Comment #2

 “… As the authors correctly emphasize, IPDMA allows for a reduction of bias introduced in meta-analyses because of the heterogeneity between studies. However, even in the reasonably large dataset of NERSH, no statistical tests could be applied because of between-study heterogeneity (p. 8). A more thorough discussion of this point is needed in the paper, I think, if the authors really want to make a convincing argument for the use of databases such as NERSH: What is needed to reduce this heterogeneity, what might be disadvantages of a homogenizing approach and how much information is necessary to enable sufficient statistical power? The authors briefly discuss some of these issues on p13, but I recommend expanding this.”

 Answer #2

 Yes, heterogeneity is a significant challenge in the data pool. Doing international and cross-cultural research we expected a significant degree of heterogeneity, and because we collected already existing datasets, we were not able to reduce the heterogeneity already present between the raw samples. Still, we know from another project/article (currently undergoing peer-review elsewhere) that we can reduce the between-study heterogeneity by grouping the physicians by medical specialty. Further stratification of the health professionals in the data pool would likely further reduce the heterogeneity making comparisons more feasible. A disadvantage of this approach would be the risk of diluting a potential effect by creating small groups. The current article is however not a meta-analysis, and we have chosen to report the complete content of the data pool here.

 Another and more long-term strategy to reduce heterogeneity would be to reduce methodological biases of sampling of future datasets. This could be done for instance by ensuring thorough cross-cultural validation of local questionnaires including qualitative feedback from health professions who completed the questionnaire, ensuring that responders are presented with explaining definitions of central terms and concepts referenced in the questionnaire, and unifying the planning and execution of the local surveys internationally.

 We have added a short discussion of this to the Limitations section of the article.

 Regarding an estimate of sample size needed to receive sufficient statistical power, for the current study this would be purely theoretical and of little practical relevance. It could however make sense to calculate such estimate before conducting future hypothesis-testing studies considering the expected variance of the selected measurements of those specific studies.

 Comment #3

 “On p4, section 'building the NERSH Data Pool' it is stated that only survey samples that weren't already included were selected. However, I wondered if this meant that any repeated-measures samples would be excluded? Though I understood toward the end of the paper that such samples were non-existent, I suggest changing the word 'samples' to 'datasets', to avoid this misunderstanding.”

 Answer #3

 No samples were repeated so there were no repeated-measures samples excluded. If we had access to any repeated-measures samples they would be of great value to us and would have been included in the data pool.

 In the section ‘Building the NERSH Data Pool’ we made two clarifications using ‘dataset’ rather than samples.

 Comment #4

 “I also wasn't fully clear on the search strategy. Though I could follow the text, I became confused when looking at the upper half of Table 1; Have you looked for studies that cited those studies by Curlin et al.? If so, why did you choose these studies as for the citation search?”

 Answer #4

 The eight articles by Curlin used in the citation search were selected by Hvidt and Kørup in 2016 for the first systematic search. It was important for us to use the exact same approach in this study. Articles were chosen because they all rest on the RSMPP and were the first articles published by Curlin in the years 2005 to 2008. Looking for publications using the RSMPP we judged it unlikely that at least one of these articles would not be referenced. Today we have executed this search strategy twice (in 2016 and 2020), both times followed by a rigorous literature search as also described in the article. Both times the literature search was not able to find further samples that we had not already found using the network and citation searches.

 I have added this elaboration to the article as well to clarify this part of the study design.

 Comment #5

 “Do I understand it correctly that Table 2 contains all of the datasets currently in the NERSH database and that the ones listed on p7 are the new ones that were added to the third version? If so, please make this more clear in the text.”

 Answer #5

 Yes, it was a bit misleading in the text. We have rephrased it to “After inclusion of the found datasets the complete data pool comprised 4,872 physicians, 1,319 nurses, 286 midwifes, and other HP occupations in smaller numbers (See Table 2 for details)”.

 Comment #6

 “Why have you chosen to only examine the data of physicians and nurses?”

 Answer #6

 We present these statistics to give a small view into the characteristics of the health professionals in data pool. Physicians and nurses comprise 85% of the observations in the data pool why they were suited for this purpose without grouping different occupations together. On the other hand, we did not want to perform any actual statistical comparison between the groups due to the heterogeneity between samples (Such analysis would require a study of its own). We decided to report these statistics to give readers a hint of the scope and potential of the data pool in relation to future studies and collaborations.

 Comment #7

 “Have I understood correctly that the exclusion criteria mentioned on p8 are missing information on gender, only containing empty answers and not being 18years of age or older? Perhaps this could be made more clear in the methods section?”

 Answer #7

 Yes, you are correct. This information is already available in the Methods section under the title ‘Inclusion and exclusion criteria’. I have added clarifications in the text.

 Comment #8

 “Finally, there are some misspellings and awkward sentences throughout the text that another careful reading should be able to get out. “

 Answer #8

 We have attended to several minor misspellings/grammatical issues through-out the manuscript.

 Comment #9

 “Overall, I really appreciate the efforts of the authors to advance research methodology in the field of religion, spirituality, and health, and I think this paper helps the field make an important step in that direction..”

 Answer #9

 Thank you.
